# Clinico-pathological and treatment-related factors influencing survival in parotid cancer

**DOI:** 10.1038/sj.bjc.6990501

**Published:** 1999-06

**Authors:** A G Renehan, E N Gleave, N J Slevin, M McGurk

**Affiliations:** Departments of Surgery and Clinical Oncology, Christie Hospital NHS Trust, Manchester, UK; Salivary Gland Service, Department of Oral and Maxillofacial Surgery, Guy's Hospital, London, UK

**Keywords:** parotid carcinoma, prognostic factors, surgery, radiotherapy

## Abstract

One hundred and three patients with primary parotid cancer treated surgically at the Christie Hospital, Manchester (1952–1992), were analysed to assess the influence on survival of prognostic and treatment-related factors. Thirty-seven patients were treated by surgery alone (SG), 66 received post-operative radiation (SG+RT). Median follow-up was 12 years, minimum 5 years. The 10-year disease-specific survival rates for stage I, II and III/IV were 96%, 61% and 17% respectively (*P* < 0.0001). The various histological types segregated into three survival patterns: low-, intermediate-and high-grade with 10-year survival rates of 93%, 41% and 50% respectively (*P* < 0.0001). On multivariate analysis, the factors influencing risk of cancer death in order of importance were: tumour size > 4 cm (*P* < 0.001), presence of nodes (*P* = 0.001), histology of adenoid cystic carcinoma (*P* = 0.01), high-tumour grade (*P* = 0.02) and perineural involvement (*P* = 0.01). Neither the extent of surgery nor the operator influenced outcome. Overall, adjuvant RT significantly reduced locoregional recurrence (SG+RT 15% vs SG 43%; *P* = 0.002) but not survival, although on subanalysis, there was a trend to improved survival with large cancers and high-grade tumours. Long-term survival is determined primarily by tumour characteristics, namely clinical stage and grade. Post-operative RT contributes significantly to locoregional control and probably confers some survival advantage in high-risk patients. © 1999 Cancer Research Campaign


*British Journal of Cancer* (1999) **80**(8), 1296–1300
© 1999 Cancer Research Campaign
Article no. bjoc.1999.0501

### Clinico-pathological and treatment-related factors influencing survival in parotid cancer

**AG Renehan[1], EN Gleave[1], NJ Slevin[2] and M McGurk[3]**

Departments of [1]Surgery and [2]Clinical Oncology, Christie Hospital NHS Trust, Manchester, UK; [3]Salivary Gland Service, Department of Oral and Maxillofacial Surgery, United Medical and Dental Schools, Guy's Hospital, London, UK

**Summary** One hundred and three patients with primary parotid cancer treated surgically at the Christie Hospital, Manchester (1952–1992), were analysed to assess the influence on survival of prognostic and treatment-related factors. Thirty-seven patients were treated by surgery alone (SG), 66 received post-operative radiation (SG+RT). Median follow-up was 12 years, minimum 5 years. The 10-year disease-specific survival rates for stage I, II and III/IV were 96%, 61% and 17% respectively ($P < 0.0001$). The various histological types segregated into three survival patterns: low-, intermediate-and high-grade with 10-year survival rates of 93%, 41% and 50% respectively ($P < 0.0001$). On multivariate analysis, the factors influencing risk of cancer death in order of importance were: tumour size > 4 cm ($P < 0.001$), presence of nodes ($P = 0.001$), histology of adenoid cystic carcinoma ($P = 0.01$), high-tumour grade ($P = 0.02$) and perineural involvement ($P = 0.01$). Neither the extent of surgery nor the operator influenced outcome. Overall, adjuvant RT significantly reduced locoregional recurrence (SG+RT 15% vs SG 43%; $P = 0.002$) but not survival, although on subanalysis, there was a trend to improved survival with large cancers and high-grade tumours. Long-term survival is determined primarily by tumour characteristics, namely clinical stage and grade. Post-operative RT contributes significantly to locoregional control and probably confers some survival advantage in high-risk patients.

**Keywords**: parotid carcinoma; prognostic factors; surgery; radiotherapy,

Carcinomas of the parotid gland are relatively uncommon malignancies (0.6 per $10^5$ population; HMSO, 1997). They are frequently characterized by a long natural history and, consequently, their study is rendered difficult both by the time taken to accrue sufficient patients for analysis and the fact that at least 10-year follow-up is required to adequately assess treatment outcome (Spiro, 1986). Prospective randomized trials are usually impractical in such circumstances. Consensus on treatment can only be obtained from analysis of large retrospective studies in which multivariate analysis is the most appropriate method of evaluation. The purpose of this study is to identify important prognostic and treatment-related factors that influence survival in parotid cancer. For purposes of comparison, factors critical to locoregional recurrence and distant metastasis will also be examined.

### PATIENTS AND METHODS

Between 1952 and 1992, a total of 825 patients with previously untreated parotid neoplasms were surgically treated at the Christie Hospital, Manchester and previously summarized by the authors (Renehan et al, 1996). A histological diagnosis of carcinoma was established in 143 patients (16%), of which 40 patients were considered incurable at diagnosis and treated palliatively. The remaining 103 formed the focus of this study. Survival data and disease status has been evaluated to 1997, such that all survivors were followed for a minimum of 5 years (median 12, range 5–32).

*Received 3 September 1998*
*Accepted 22 January 1999*

*Correspondence to:* M McGurk

There were 52 males (median age: 55; range 9–85 years) and 51 females (median age: 62; range 10–92 years). The median duration of symptoms was 10 (range 1–300) months. Tumours frequently presented as clinically 'benign' lumps with frank malignant features seen in only 42 (41%) patients (indurated tumour, 39; facial nerve palsy, 12; including nine with both). Cervical lymphadenopathy was uncommon at presentation (8%) and notably was not documented in patients with adenoid cystic carcinoma (Fisher's exact test; $P < 0.0001$).

Tumours were staged retrospectively in accordance with AJCC (Fleming et al, 1997). Histological classification was reviewed in the mid-1970s and updated in line with the criteria of WHO (Seifert and Sobin, 1991). Tumour types were assigned to three grades representing different levels of biological behaviour. Mucoepidermoid and adenocarcinoma were subclassified into low-and high-grade based on histological features, while the remaining tumour types were assigned as reflected by their natural history (Table 1). This clinical grading system is similar to those proposed by the AFIP (Ellis et al, 1991) and other major institutes (Spiro, 1986; Kane et al, 1991).

All patients were treated surgically by two consecutive surgeons ensuring continuity in management (late WAB Nicholson 1956–1972; ENG 1973–1992). Twenty-four (23%) underwent local extracapsular dissection (McGurk et al, 1996), 45 (44%) formal parotidectomy with nerve identification (superficial, 34; total, 11) and 34 (33%) a more radical approach (partial nerve sacrifice, 12; total nerve sacrifice, six; extended parotidectomy, 16). Radical neck dissection was performed in eight patients, all with clinically palpable nodes.

Sixty-six (64%) patients received post-operative radiotherapy (RT). In the early part of the series, five patients were treated (as was routine at that time) with interstitial therapy (single plane and

**Table 1** Clinical and histological characteristics by grade

| | Grade | | | |
|---|---|---|---|---|
| | Low ($n$ = 41) | Intermediate ($n$ = 23) | High ($n$ = 39) | Total ($n$ = 103) |
| Age | | | | |
| Median (years) | 50 | 60 | 66 | 59 |
| Range (years) | 9–85 | 31–80 | 26–92 | 9–92 |
| < 40 years | 15 | 1 | 3 | 19 (18) |
| Stage (AJCC 5th ed.) | | | | |
| I (T1N0M0, T2N0M0) | 22 | 5 | 5 | 32 (31) |
| II (T3N0M0) | 14 | 14 | 20 | 48 (47) |
| III/IV (T4N0M0, any TN1M0) | 5 | 4 | 14 | 23 (22) |
| Histological type | | | | |
| Acinic cell ca. | 9 | – | – | 9 (9) |
| Mucoepidermoid ca. | 22 | – | 1 | 23 (22) |
| Basal cell adenocarcinoma | 1 | – | – | 1 |
| Papillary cystadenocarcinoma | 4 | – | – | 4 (4) |
| Mucinous adenocarcinoma | 1 | – | – | 1 |
| Adenocarcinoma, NOS | 4 | – | 7 | 11 (11) |
| Adenoid cystic carcinoma | – | 22 | – | 22 (21) |
| Epithelial-myoepithelial ca. | – | 1 | – | 1 |
| Ca. ex-pleomorphic adenoma | – | – | 14 | 14 (14) |
| Squamous cell carcinoma | – | – | 5 | 5 (5) |
| Undifferentiated carcinoma | – | – | 12 | 12 (12) |

Values in parentheses are percentages. NOS: not otherwise specified.

V-implants) which typically delivered 60 Gy in 7 days. Sixty-one patients were treated with once daily megavoltage radiation (4 MeV: 50–55 Gy: 15–16 fractions: median 21 days). The indications were: positive (12 of 16 had RT) or equivocal (28 of 34) margins, T size > 4 cm (38 of 52), high-grade (28 of 39), adenoid cystic histology (12 of 22) and perineural involvement (eight of 12), and many patients had more than one indication. An inclined plane was used to avoid exit beams irradiating the eyes and the techniques grouped as single field (three patients), wedge pair (18 patients) and wedge three field (40 patients). The median time to the start of radiation was 33 days post-operatively.

### Statistical analysis

Survival curves were calculated by the Kaplan–Meier method. Distributions were compared using the log-rank test and the Cox proportional hazards regression model used to identify independent determinants of survival. The percentages of patients with locoregional recurrences and distant metastases were analysed as simple proportions as the length of follow-up and ways of censoring were similar.

### RESULTS

### Prognostic factors

### *Survival*

The overall 5-, 10- and 15-year disease-specific survival rates were 78%, 65% and 63% respectively. There were 33 cancer deaths; 20 with distant metastases (DM) only, eight with locoregional recurrence (LRR) only and five with both DM and LRR. There were no treatment-related deaths.

The factors influencing survival on univariate analysis, in order of significance, were: tumour size ($P < 0.0001$), palpable cervical nodes ($P < 0.0001$), histological type ($P = 0.003$), patient's age

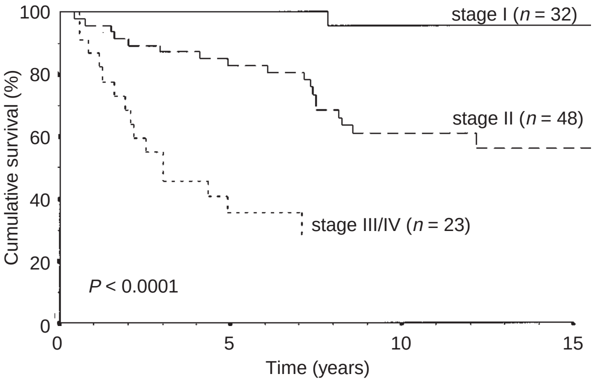

**Figure 1** Survival by clinical staging

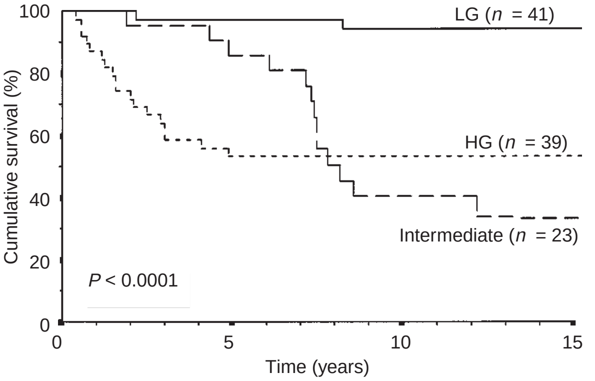

**Figure 2** Survival by tumour grade. LG = low grade; HG = high grade

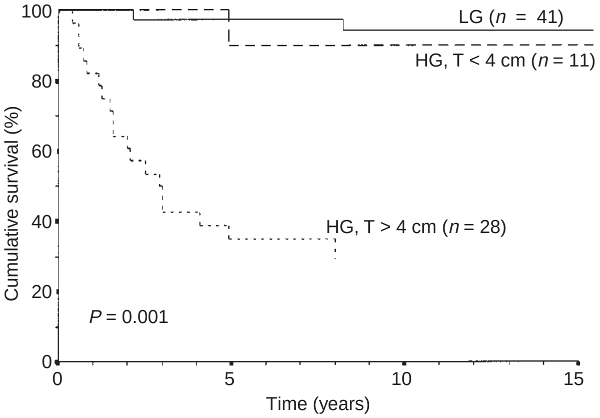

**Figure 3** Survival in low and high-grade tumours according to tumour size. LG, low-grade; HG, high-grade. For clarity, all LG tumours (large low grade = 9) are considered together

($P = 0.003$), microscopic disease in para-parotid nodes ($P = 0.004$), positive or close microscopic tumour margin ($P = 0.01$), perineural involvement ($P = 0.02$), local extension with and without facial nerve palsy ($P = 0.03$) and duration of symptoms ($P = 0.04$).

Clinical stage (a composite of T size, local extension and nodal status) was very predictive for survival; the 10-year disease-specific survival rates for stage I, II and III/IV were 96%, 61% and 17% respectively (Figure 1). The 10-year survival for patients with low-, intermediate- and high-grade cancers was 91%, 41% and

© 1999 Cancer Research Campaign 

**Table 2** Multivariate analyses for survival, locoregional recurrence and distant metastasis

| Variable | | Survival | | | Locoregional recurrence | | | Distant metastasis | | |
|---|---|---|---|---|---|---|---|---|---|---|
| | | *P*-value[a] | RR | 95% CI | *P*-value | RR | 95% CI | *P*-value | RR | 95% CI |
| T size[b] | (≥ 4 cm vs < 4 cm) | < 0.001 | 8.17 | 2.4–27.5 | 0.01 | 3.09 | 1.3–7.3 | 0.005 | 8.49 | 1.9–37 |
| Cervical node | (yes vs no) | 0.001 | 7.57 | 2.7–21.2 | NS | | | 0.01 | 4.49 | 1.3–15 |
| Local extension | (yes vs no) | NS | | | NS | | | 0.02 | 2.87 | 1.1–7.2 |
| Grade[c] | (ADCC vs low) | 0.01 | 7.21 | 1.6–33.1 | NS | | | 0.05 | 7.98 | 1.0–36 |
| | (high vs low) | 0.02 | 6.12 | 1.4–27.6 | NS | | | 0.05 | 7.64 | 1.0–30 |
| Perineural invasion[d] | (yes vs no) | 0.01 | 3.74 | 1.3–10.6 | NS | | | NS | | |
| Post-operative RT | (yes vs no) | NS | | | 0.003 | 0.24 | 0.1–0.5 | NS | | |

RR, relative risk; CI, confidence interval; RT, radiotherapy; ADCC, adenoid cystic carcinoma; NS, not significant. [a]Likelihood ratio test using forward stepwise method. [B]Separate model: stage substituted for size, nodal status and local extension: Stage II, RR, 8.37, *P* = 0.04, Stage III/IV, RR, 24.1, *P* = 0.002. [c]Non-binary variables treated as categorical to avoid contamination of the *P*-value caused by searching for the most informative cut-point. [d]Based on a model of 91 patients due to incomplete data.

**Table 3** Extent of surgery and outcome

| Extent of surgery | Positive/equivocal margin[a] | Post-operative RT | Local failure | 10-year survival (%) |
|---|---|---|---|---|
| All tumours | | | | |
| a)Nerve preservation | | | | |
| Local dissection | 10/20 (50) | 13/24 (54) | 5/24 (21) | 68 |
| Formal parotidectomy | 23/41 (56) | 28/45 (62) | 9/45 (20) | 78 |
| b)Nerve sacrifice | 17/30 (57) | 25/34 (74) | 4/34 (12) | 45[b] |
| Stage I (mobile tumours < 4 cm, *n* = 32) | | | | |
| a) Nerve preservation | | | | |
| Local dissection | 4/9 (44) | 6/12 (50) | 1/12 (8) | 100 |
| Formal parotidectomy | 7/18 (39) | 10/19 (53) | 5/19 (26) | 94 |
| b) Nerve sacrifice | 1/1 | 1/1 | 0 | |

[a]exact histological information on margin status was unavailable in 4 patients. [b]survival for radical surgery vs nerve preserving surgery: 45% vs 74% at 10 years, *P* = 0.003.

50% respectively (Figure 2). Tumour size was more important than tumour grade, for in small tumours, the prognosis was good for both low-and high-grade tumours (100% vs 96% at 10 years) compared to a markedly worse outcome for high-versus low-grade (35% vs 75% at 10 years) in larger tumours (Figure 3).

The multivariate analysis revealed that tumour size, presence of cervical nodes, clinical grade and perineural involvement were independent predictors for survival (Table 2).

### *Locoregional recurrence*

The LRR rate was 25% with a median interval following initial surgery of 30 months (range 5–168). Of the 26 patients with recurrence, 18 were at the primary site, nine were nodal, including one patient who relapsed at both sites. All but one nodal metastasis occurred on the ipsilateral side at AAOHNS neck levels I, II and III (Robbins et al, 1991). No single factor was predictive of LRR by univariate analysis, probably due to the confounding effect of adjuvant RT, but multivariate analysis demonstrated that tumour size (negatively) and adjuvant radiation (positively) were independently important.

### *Distant metastasis*

Twenty-five (24%) patients had clinically recognizable distant metastases (DM). The commonest sites were: lung (*n* = 17), bone (*n* = 6) and brain (*n* = 4). DM were influenced, on univariate analysis, by tumour size (T1, 0%; T2, 5%; T3, 38%; T4, 73%; *P* < 0.0001) and grade (low, 2%; intermediate, 44%; high, 36%; *P* < 0.001) and, on multivariate analysis, were best predicted by tumour size, presence of cervical nodes, local extension and grade. Despite apparent local cure in 77 patients, 20 (26%) patients still developed DM, suggesting that in many patients microscopic dissemination had already occurred at presentation.

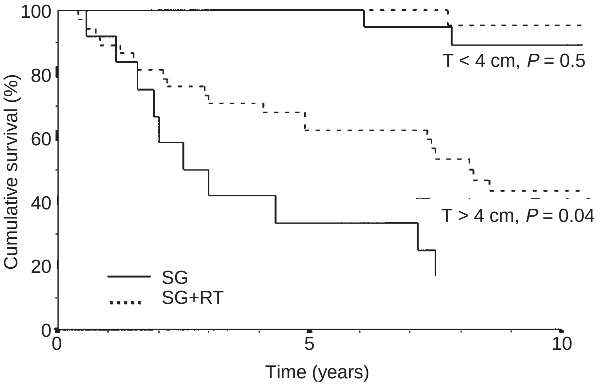

**Figure 4** Comparison of survival in patients treated with surgery alone versus combined therapy according to tumour size. T, tumour; SG, surgery alone; SG+RT, surgery with adjuvant radiation. Numbers in lower half of graph are number of patients per group at time zero

 *© 1999 Cancer Research Campaign*

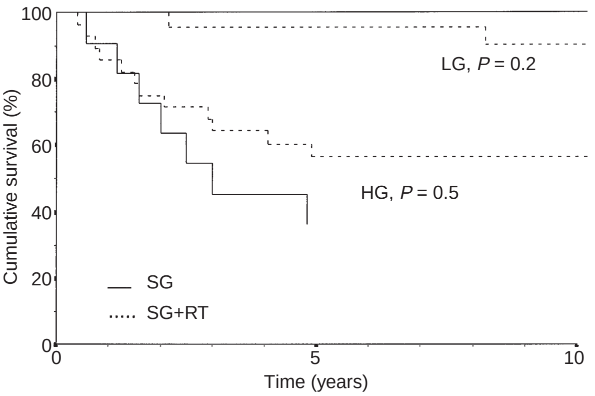

**Figure 5** Comparison of survival in patients treated with surgery alone versus combined therapy according to tumour grade. LG, low-grade; HG, high-grade; SG, surgery alone: SG+RT, surgery with adjuvant radiation. Numbers in lower half of graph are number of patients per group at time zero

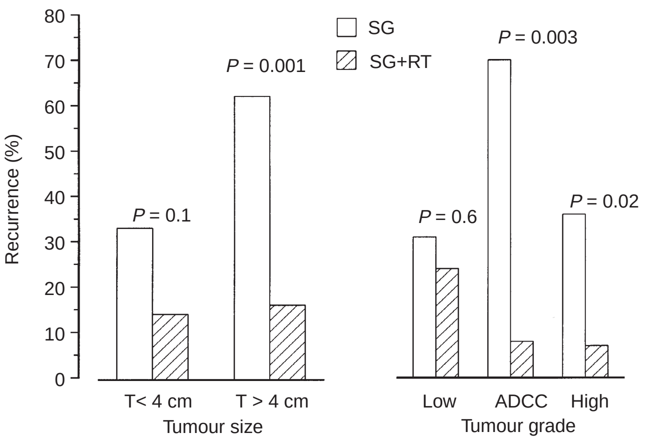

**Figure 6** Comparison of locoregional recurrence rates in patients treated by surgery alone versus combined therapy considered by tumour size and tumour grade. Numbers below horizontal line are number of patients per group. ADCC denotes adenoid cuptic

### Treatment-related factors

### *Extent of surgery*

Not surprisingly, survival was worse for patients undergoing radical surgery versus nerve-preserving surgery (45% vs 74% at 10 years, $P = 0.003$) but the extent of surgery had no influence on survival in a multivariate model taking T size, grade, adjuvant radiotherapy and surgeon into consideration. Similarly, there was no difference in outcome between the two surgeons when adjusted for case-mix.

Subanalysis of the surgical techniques used in 32 stage I cancers which presented as 'clinically benign' demonstrated no difference in outcome between extracapsular dissection and superficial parotidectomy (Table 3). Local failures were more frequent following formal parotidectomy (26% vs 8%, $P = 0.4$), although 10-year survival rates differed little (100% vs 94%, $P = 0.48$)

### *Combined therapy versus surgery alone*

For the whole group, there was no difference in survival between combined therapy versus surgery alone (5- and 10-year survival rates for SG+RT were 78%, 67% vs SG, 77%, 63%, $P = 0.83$). The Cox analysis confirmed that survival was unaffected by post-operative RT but when outcome was subanalysed by tumour size and grade, adjuvant RT provided some survival benefit in tumours > 4 cm (SG+RT, 63% vs SG, 33% at 5 years) (Figure 4) and high-grade disease (SG+RT, 56% vs SG, 45% at 5 years) (Figure 5).

Adjuvant radiotherapy significantly reduced LRR (SG+RT, 15% vs SG, 43%, $P = 0.002$), which was confirmed in the Cox analysis. All patient subgroups benefited from adjuvant radiation, though this was most evident in patients with tumours > 4 cm, a histology of adenoid cystic carcinoma and high-grade disease (Figure 6).

### DISCUSSION

This study confirms that long-term survival in parotid cancer is determined primarily by tumour characteristics, mainly clinical stage and tumour grade (Calearo and Pastore, 1995), and that post-operative radiation significantly improves locoregional control but not survival (Jackson et al, 1983; Theriault and Fitzpatrick, 1986, Spiro et al, 1989). Three additional aspects of the clinical course and treatment of this malignancy have been identified: First, from a treatment perspective, histological classification can be simplified into three categories; second, a conservative surgical approach to small apparently 'benign' parotid cancers does not compromise local control and survival; and third, post-operative radiation may offer some survival advantage in large and high-grade tumours.

In terms of prognosis, tumour size was the most important variable for no cancer deaths occurred among patients with tumours < 2 cm, but all patients with tumours > 6 cm died of disease. Nodal metastasis was an uncommon event but when present it was an important adverse prognostic factor (Spiro et al, 1993). Facial nerve palsy is another poor prognostic sign, though it did not indicate incurable disease as six of 12 patients experienced long-term disease-free survival. In contradiction to other reports (Frankenthaler et al, 1991; Poulson et al, 1992), age was not an independent predictor of survival, though notably, young patients tended to have low-grade tumours (79%) and there were no cancer-related deaths in patients under 40 years.

Based on survival analysis, the various different histological types segregated into three clinical patterns; low-grade (indolent), intermediate grade and high-grade (aggressive) tumours. The natural history of low-grade cancers (acinic cell, low-grade mucoepidermoid, papillary cystadenocarcinoma, basal cell adenocarcinoma) is for long-term disease-free survival, while high-grade cancers (carcinoma ex-pleomorphic adenoma, high-grade mucoepidermoid, squamous cell, undifferentiated) behave more like squamous cell carcinomas of the upper aerodigestive tract. Intermediate grade cancers (predominantly adenoid cystic carcinoma) show a pattern of progressive failure through distant metastases. The latter observation supports Spiro's assertion (Spiro et al, 1989) that subclassification of adenoid cystic carcinoma into histomorphological patterns (solid, trabular, cribiform) probably matters little to long-term survival.

The influence of surgical procedure on outcome was difficult to disentangle from confounding factors such as adjuvant radiotherapy though the extent of surgery did not effect outcome in either the univariate or multivariate analysis. The failure to identify a difference may have resulted as the treatment selected was appropriate for the individual tumour. Contrary to expectations, extracapsular dissection did not effect outcome in Stage I tumours,

*© 1999 Cancer Research Campaign*

the inference being that small mobile parotid cancers do not require wide resection (Leverstein et al, 1998). This in practice is the approach adopted by most surgeons since these tumours present as clinically benign lumps and are treated as such (McGurk et al, 1995).

In general, survival from parotid cancer was unaffected by post-operative RT, a conclusion also reached by others (Spiro et al, 1989). Accepting some unfavourable selection bias in patients receiving combined therapy, the expectation was for a worse outcome in these patients. However, this was not the case but rather a modest (and probably real) survival benefit was identified with adjuvant RT in patients with tumours > 4 cm. This confirms similar observations made by Armstrong et al (1990), and mirrors the survival benefits of adjuvant RT seen in patients with high-risk breast cancer (Overgaard et al, 1997). The influence on survival of adjuvant RT in high-grade tumours is more complex as smaller survival advantages were seen in this series and others (Armstrong et al, 1990; Frankenthaler et al, 1991). Some of the beneficial effect of adjuvant therapy may simply reflect tumour size as small, high-grade tumours had a good prognosis (96% at 5 years) (Spiro and Huvos, 1992).

The overall incidence of DM (24%) was similar to that reported elsewhere and best predicted by tumour size, local extension, nodal status and grade (Gallo et al, 1997). This was unaffected by post-operative RT reaffirming improvement in locoregional control remains its main role in the treatment of parotid cancer (Sykes et al, 1995). Some 80% of DM occurred despite locoregional control, which suggests both early dissemination of disease and the need to develop new effective systemic treatment strategies.

The current histological classification of salivary gland cancer has over-influenced thinking on the treatment of salivary tumours (McGurk et al, 1995). The present results emphasize the importance of clinical factors rather than histology in determining treatment results.

### DEDICATION

This manuscript is dedicated to Mr E Neville Gleave who died during the preparation of this paper.

### REFERENCES

Armstrong JG, Harrison LB, Spiro RH, Fass DE, Strong EW and Fuks ZY (1990) Malignant tumors of major salivary gland origin. A matched-pair analysis of the role of combined surgery and postoperative radiotherapy. *Arch Otolaryngol Head Neck Surg* **116**: 290–293

Calearo C and Pastore A (1995) Parotid carcinoma. In: *Prognostic Factors in cancer, UICC*, Hermanek P, Gospodarowie M, Henson D, Hutter R and Sobins L (eds), pp. 23–27. Springer-Verlag: Berlin

Ellis G, Auclair P and Gnepp D (1991) *Surgical Pathology of the Salivary Glands*. WB Saunders Co: Philadelphia

Fleming I, Cooper J, Henson D, Hutter R, Kennedy B and Murphy G (1997) *AJCC Cancer Staging Manual*. Lippincott-Raven: Philadelphia

Frankenthaler RA, Luna MA, Lee SS, Ang KK, Byers RM, Guillamondegui OM, Wolf P and Goepfert H (1991) Prognostic variables in parotid gland cancer. *Arch Otolaryngol Head Neck Surg* **117**: 1251–1256

Gallo, O, Franchi, A, Bottai, GV, Fini Storchi, I, Tesi, G and Boddi, V (1997) Risk factors for distant metastases from carcinoma of the parotid gland. *Cancer* **80**: 844–851

HMSO (1997) *Cancer Statistics Registrations 1991*. Series MB1 no. 24. pp24. Office for National Statistics: London

Jackson G, Luna M and Byers R (1983) Results of surgery alone and surgery combined with postoperative radiotherapy in the treatment of cancer of the parotid gland. *Am J Surg* **146**, 697–700

Kane, WJ, McCaffrey TV, Olsen KD and Lewis JE (1991) Primary parotid malignancies. A clinical and pathologic review. *Arch Otolaryngol Head Neck Surg* **117**, 307–315

Leverstein H, van der Waal JE, Tiwari RM, Tobi H, van der Waal I, Mehta DM and Snow GB (1998) Malignant epithelial parotoid gland tumours: analysis and results in 65 previously untreated patients. *Br J Surg* **85**: 1267–1272

McGurk M, Renehan A, Gleave EN and Hancock BD (1996) Clinical significance of the tumour capsule in the treatment of parotid plemorphic adenomas. *Br J Surg* **83**, 1747–1749

McGurk M, Williams R and Calman F (1995) A Clinical Approach to Malignant Tumours. In: *Colour Atlas and Text of Salivary Glands: Diseases, Disorders and Surgery*, de Norman J and McGurk M (eds), pp. 181–196. Mosby-Wolf: London

Overgaard M, Hansen PS, Overgaard J, Rose C, Andersson M, Bach F, Kjaer M, Gadeberg CC, Mouridsen HT, Jensen MB and Zedeler K (1997) Postoperative radiotherapy in high-risk premenopausal women with breast cancer who receive adjuvant chemotherapy. Danish Breast Cancer Cooperative Group 82b Trial [see comments]. *N Engl J Med* **337**, 949–955

Renehan A, Gleave EN, Hancock BD, Smith P and McGurk M (1996) Long-term follow-up of over 1000 patients with salivary gland tumours treated in a single centre. *Br J Surg* **83**, 1750–1754

Robbins K, JE, M, Wolfe G, Levine P, RB, S and Pruet C (1991) Standardizing neck dissection terminology. Official report of the Academy's committee for Head and Neck Surgery and Oncology. *Arch Otolaryngol Head Neck Surg* **117**, 601–605

Seifert G and Sobin L (1991) *WHO Histological Typing of Salivary Gland Tumours*. Springer-Verlag: Berlin.

Spiro RH (1986) Salivary neoplasms: overview of a 35-year experience with 2,807 patients. *Head Neck Surg* **8**, 177–184

Spiro RH, Armstrong J, Harrison L, Geller NL, Lin S-Y and Strong EW (1989) Carcinoma of major salivary glands. *Recent Trends Arch Otolaryngol Head Neck Surg* **115**: 316–321

Spiro RH and Huvos AG (1992) Stage means more than grade in adenoid cystic carcinoma. *Am J Surg* **164**, 623–628

Spiro IJ, Wang CC and Montgomery WW (1993) Carcinoma of the parotid gland. Analysis of treatment results and patterns of failure after combined surgery and radiation therapy. *Cancer* **71**, 2699–2705

Sykes AJ, Logue JP, Slevin NJ and Gupta NK (1995) An analysis of radiotherapy in the management of 104 patients with parotid carcinoma. *Clin Oncol R Coll Radiol* **7**, 16–20

Theriault C and Fitzpatrick PJ (1986) Malignant parotid tumors. Prognostic factors and optimum treatment. *Am J Clin Oncol* **9**, 510–516

 © 1999 Cancer Research Campaign
